# Ribociclib and palbociclib-induced erythema multiforme: a case report

**DOI:** 10.1093/omcr/omac116

**Published:** 2022-11-24

**Authors:** Eleni Vrana, Stella Mylona, Mattheos Bobos, Loukas Kontovinis, Konstantinos Papazisis

**Affiliations:** “Euromedica” General Clinic, Oncology Department, 54645 Thessaloniki, Greece; Dermatologist, 55133 Thessaloniki, Greece; Pathology, Microdiagnostics LP, Thessaloniki, Greece; “Euromedica” General Clinic, Oncology Department, 54645 Thessaloniki, Greece; “Euromedica” General Clinic, Oncology Department, 54645 Thessaloniki, Greece

## Abstract

Cyclin-dependent kinase 4/6 inhibitors (CKIs), ribociclib, palbocilb and abemaciclib, have been approved in combination with endocrine therapy for the treatment of hormone receptor-positive and human epidermal growth factor 2-negative advanced or metastatic breast cancer. Severe dermatological adverse events are rare with these agents; however, they require direct recognition and management in order not to become life-threatening. Erythema multiforme (EM) belongs to a dermatopathic spectrum that includes immune-mediated, widespread hypersensitivity reaction, which occurs with varying degrees of severity and affects the skin and/or the mucosa. We hereby present a case of ribociclib- and palbociclib-related EM. We sought to report this case given the implication of two agents from the same drug class in EM onset. We also aim to emphasize the breadth of mechanisms of actions of CKIs, with an impingement in the immune system as well, and the importance of promptly identifying and handling such skin toxicities.

## INTRODUCTION

Erythema multiforme (EM) comprises an acute, immune-mediated, self-limited and sometimes recurring condition, which is considered a type IV hypersensitivity reaction associated with certain infections, medications and other various triggers [1]. It affects the skin and/or mucous membranes, depending on its extent (minor or major), and its classic lesion is called ‘target or iris lesion’. Its lesions are typically initiated from the extremities with a predilection for the extensor surfaces, and they spread centripetally, even though patients’ trunk is usually less affected [2].

Cyclin-dependent kinases (CDKs) 4/6 interacting with cyclin D pose an essential role in cell division and proliferation, inducing the transition from G1 to S phase of the cell cycle. Thus, CDK4/6 inhibitors (CKIs) block cell cycle progression. CKIs, including palbociclib, ribociclib and abemaciclib, are amongst the newest targeted therapies approved for locally advanced or metastatic ﻿hormone receptor (HR)-positive and human epidermal growth factor receptor 2 (HER2)-negative breast cancer (BC). Their most common adverse events (AEs) are haematologic and gastrointestinal [3]. Cutaneous toxicities have been reported to an ~15% of total AEs with CKIs and are mostly grade 1 [4]. We hereby describe the case of a female patient who developed grade 2 EM while treated with ribociclib and later on with palbociclib.

## CASE REPORT

A 50-year-old premenopausal female was diagnosed with HR-positive and HER2-negative invasive ductal carcinoma. She underwent a right mastectomy and axillary lymph node (LN) dissection, where 2 out of the 10 removed LNs were infiltrated. Adjuvant chemotherapy and hormonal therapy followed for 5 years, with tamoxifen and luteinizing hormone-releasing hormone analogue.

Fourteen years later, the patient complained about swelling in her right arm. On physical examination, swelling and palpable masses in the field of the preceded mastectomy and axilla, with infiltration of the overlying axillary skin, were found. The following imaging showed an 18-mm lesion on the right pectoralis major and the conterminous axilla, without pathological axillary LNs. The core biopsy from the lesion of the right axillary region confirmed the recrudescence of BC. The requested chest and abdominal computed tomography revealed multiple lung nodules (<1 cm). The patient was commenced on letrozole and ribociclib.

**Figure 1:**
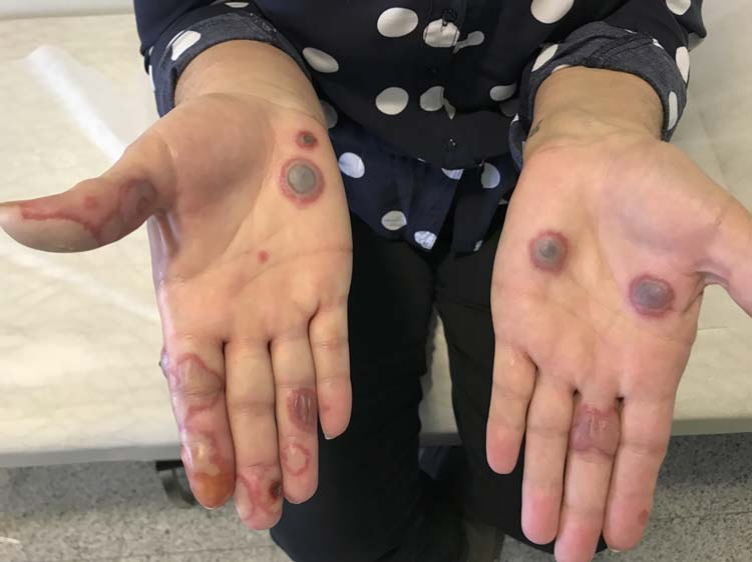
First eruption of EM-like skin lesions in palms, after treatment with letrozole and ribociclib.

Almost 6 weeks later, she complained about a skin rash with a burning and itching sensation in both her upper extremities. On physical examination, erythematous, pink-to-violaceous, tender, annular papules with dusky centres and bullae were observed on her hands bilaterally (Fig. 1). According to the patient, the lesions erupted 1 month after the initiation of the therapy and got worse over the last 2 weeks. The oral cavity was unaffected.

Regarding her medical history, she suffered from Hashimoto thyroiditis and arterial hypertension. No use of new medications or other substances was reported.

The lesions had the typical appearance of EM. However, at this first evaluation, skin biopsies were not obtained. Given the suspicion of ribociclib-mediated skin toxicity, the permanent discontinuation of ribociclib was decided. The patient continued receiving letrozole, and the rash gradually abated. A month later, the CKI palbociclib was added to the treatment.

**Figure 2:**
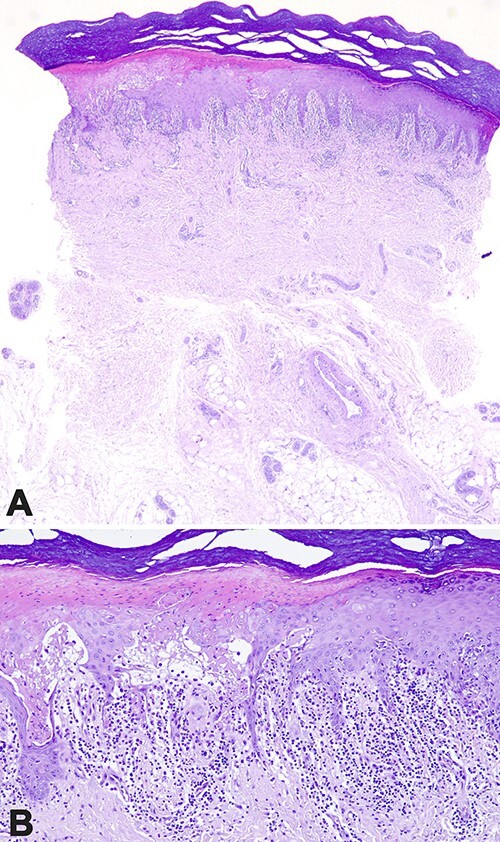
(**A**) skin punch biopsy form the hand showing lichenoid infiltrate rich in lymphocytes in the upper dermis, epidermal and focal sub-epidermal vesiculation. (**B**) The marked basal cell hydropic degeneration and apoptosis resulted in epidermal vesiculation. (A and B) Haematoxylin and eosin stain. Magnification: (A) ×40, (B) ×100.

After the first cycle of palbociclib, the patient presented grade 3 neutropenia and recurrence of the skin rash. Again, she had tender, annular papules with dusky centres and bullae with the ‘bull’s eye’ appearance. A complete physical examination revealed an aphthous lesion in the hard palate. The skin punch biopsy was obtained from the lesion on the fourth finger due to its typical ‘bull’s eye’ appearance, adequate size and easy access. It showed epidermal and subepidermal vesiculation, apoptotic keratinocytes in all levels of the epidermis and moderate lymphohistiocytic infiltration associated with lymphocytic exocytosis and satellite cell necrosis as well as extravasation of red blood cells in the upper dermis (Fig. 2). Melanin incontinence or neutrophils were not found in the infiltrate. These features confirmed the drug-mediated lichenoid skin reaction with characteristics of EM. The definite discontinuation of the CKIs was decided, and the patient was continued on letrozole. All lesions subsided within the next month. She remains in remission from BC (progression-free survival of 2 years) and without EM recurrence.

## DISCUSSION

The pathobiology of EM relies on the immune-mediated response to an inciting event. Infections constitute the trigger in ~90% of cases, with herpes simplex virus (HSV) being the most common precipitator factor [1, 2]. In contrast to viral-associated EM, where the effector cytokine is interferon-γ (IFN-γ) [5], cases of drug-induced EM are associated with tumour necrosis factor-a, perforin and granzyme B, which cause the epidermal destruction seen in the disease [1]. In cases of drug-induced EM, lesions appear within 72 hours of drug exposure but occasionally erupt over 1–2 weeks. Duration from onset to healing, typically without complications, is <4 weeks, usually about 2 weeks [6]. The diagnosis of EM is predominantly clinical, based on the patient’s history and physical examination. When the clinical picture is clear, a biopsy is not required because its findings are not specific to EM. In atypical presentation or recurrent EM without documented HSV infection, histopathological features may pose the main role in the differential diagnosis of EM from other entities that mimic its onset, such as urticaria, Stevens–Johnson syndrome, etc. [2] The treatment of EM depends on the underlying aetiology and the disease severity. The main aim is to remove the impelling factor. In the case of a drug-induced EM, this entails the discontinuation of the responsible medication [1].

The mechanism of action of CKIs is manifold. Apart from obstructing cell cycle proliferation, they are capable of upregulating major histocompatibility complex class I and enhancing antigen presentation [7]. Moreover, they increase proinflammatory cytokines, including IFN-γ, programmed death-ligand 1 expression and infiltration of the effector T cells in the tumour microenvironment [7, 8]. Last but not least, CKIs selectively deplete the T-regulatory population by suppressing their proliferation, decreasing their effect in subduing immune response simultaneously [7].

CKIs are generally considered safe, with manageable side effects. The most frequently reported amongst them include haematologic and gastrointestinal toxicities, especially neutropenia with palbociclib and ribociclib and diarrhoea with abemaciclib [3]. Skin reactions are also included in the spectrum of their AEs, yet they are usually considered mild and do not warrant a definite cessation of them [9]. However, more severe skin toxicities, such as Stevens–Johnson syndrome, have been reported in the literature [10].

Herein, we have presented a case of CKI-induced EM. In our case, EM erupted later than typically reported after drug exposure and occurred while on treatment both with ribociclib and palbociclib; thus, EM constituted a drug class-observed AE. We would like to emphasize that a multidisciplinary approach to oncological patients, as well as early recognition and sufficient management of skin toxicities, are mandatory in order to prevent lethal conditions and improve the quality of life of this population.
